# The assessment of recalled parental rearing behavior and its relationship to life satisfaction and interpersonal problems: a general population study

**DOI:** 10.1186/1471-2288-9-17

**Published:** 2009-03-06

**Authors:** Katja Petrowski, Hendrik Berth, Silke Schmidt, Jörg Schumacher, Andreas Hinz, Elmar Brähler

**Affiliations:** 1 Dresden University of Technology, Department of Psychotherapy and Psychosomatic Medicine, Fetscherstr. 74, D-01307 Dresden, Germany; 2 Dresden University of Technology, Department of Medical Psychology and Medical Sociology, Fetscherstr. 74, D-01307 Dresden, Germany; 3 Universitätsklinikum Hamburg-Eppendorf, Department of Medical Psychology and Medical Sociology, Martinistr. 52, D-20246 Hamburg, Germany; 4 University of Jena, Department of Medical Psychology, Stoystr. 3, D-07740 Jena, Germany; 5 University of Leipzig, Department of Medical Psychology and Medical Sociology, Phillip-Rosenthal-Str.55, D-04103 Leipzig, Germany

## Abstract

**Background:**

Parental rearing behavior is a significant etiological factor for the vulnerability of psychopathology and has been an issue of clinical research for a long time. For this scope instruments are important who asses economically recalled parental rearing behavior in a clinical practice. Therefore, a short German instrument for the assessment of the recalled parental rearing behavior Fragebogen zum erinnerten elterlichen Erziehungsverhalten (FEE) was psychometrically evaluated [Recalled Parental Rearing Behavior].

**Methods:**

This questionnaire was evaluated in a representative population sample (N = 2.948) in Germany which included 44.2% male and 55.8% female persons with a mean age of M = 47.35 (SD = 17.10, range = 18–92). For the content evaluation of the FEE the Life Satisfaction Questionnaire (FLZ) and the Inventory of Interpersonal Problems (IIP) was filled out by the participants.

**Results:**

The FEE scales yielded a good to satisfactory internal consistency and split-half reliability. Its three factors (rejection/punishment, emotional warmth, control/overprotection) correlated positively with most of the areas of life satisfaction. Furthermore, positive associations between interpersonal problems and parental rejection and control could be identified.

**Conclusion:**

The FEE is a short, reliable and valid instrument that can be applied in the clinical practice. In addition, the data proved an association between recalled parental rearing behavior, life satisfaction and interpersonal problems conform to the literature. Finally, specific problems with the retrospective assessment of parental rearing behavior were addressed as well.

## Background

The impact of parental rearing behavior on child development has been an issue of clinical research for a long time. Perceived parental rearing practices were emphasized as a significant etiological factor in a vulnerability model of psychopathology [1] and connected to a child's general psycho-social development as well as to the social problems of children [2]. Subjects who reported having had supportive, non-rejecting and non-overinvolved parents showed higher psychological adjustment, less social alienation and more life satisfaction [3,2]. However, age and gender moderated these effects as older individuals idealized their parents' child rearing behavior more than younger ones did [4]. On the whole, male subjects reported a more rejecting parental rearing behavior than their female counterparts did [5].

In clinical research and in retrospective studies most of the empirical results were obtained with the help of two questionnaires: The first one is the Parental Bonding Instrument (PBI [6]) and its clinical version, the Measure of Parenting Style (MOPS [7]). The PBI was comprised of the two dimensions "care" and "control", and the MOPS consisted of an additional third dimension of "parental abuse" (retest-reliability = .63 to .76 [6]). The second one was the Egna Minnen Beträffande Uppfostran (EMBU [8] [Own Memories of Child Rearing Experiences] and yielded separately a three-factorial structure with the dimensions "rejection/punishment", "emotional warmth", and "control/overprotection" for the mother as well as the father [9]. The long version of the EMBU displayed an internal reliability of < .70 [9] and the short version an internal reliability of > .72 [10].

Both questionnaires (EMBU, PBI) exist as long versions in German and show good psychometric properties. Economical short versions for clinical application do exist in English and German exclusively for the EMBU. The German short version of the EMBU, the Fragebogen zum erinnerten elterlichen Erziehungsverhalten (FEE [11]) [Recalled Parental Rearing Behavior], was already implemented in different studies examining the perceived parental rearing behavior in siblings, in clinical samples in respect to attachment and relationship characteristics [12-16]. However, the psychometric properties of this German FEE short version had not yet been specified in a representative sample. Therefore, the purpose of this study was to evaluate the psychometric properties of this short version based on a representative sample.

The first objective of this study was the specification of internal reliability of this German FEE short version based on a representative German sample. This questionnaire might show a similar range of internal reliability indices as published for the different English EMBU versions.

The second objective of this study was the evaluation of the construct validity of this German FEE short version. Starting from the assumption that similar psychometric properties and results as based on the English version are replicable, the male subjects may state more rejection in parental rearing behavior than female ones. Whereby, the older subjects might idealize the parental rearing behavior more than the younger subjects did. Furthermore, non-supportive and rejecting parental rearing behavior may be associated with more interpersonal problems and less life satisfaction.

## Methods

### Subjects

The sample consisted of 2,948 subjects constituting a representative sample of the German population interviewed by a demographic consulting company (USUMA, Berlin) in 1994. The selection of the households by the random-route-procedure was based on the register of the political elections of 1994. The randomly selected household member had to be over 18 and a native speaker. The subjects were asked to fill out an extensive questionnaire. The response rate of this examination was 68%. This sample was representative for the main socio-demographic data of the German population (age, gender, city, county and education). The mean age of this sample was M = 47.35 (SD = 17.10, range = 18–92) with 44.2% male subjects and 55.8% female subjects.

The study followed the ethical guidelines of the "German professional institutions of social researcher" [Arbeitskreis Deutscher Markt- und Sozialforschungsinstitute e.V. (ADM), Arbeitsgemeinschaft Sozialwissenschaftlicher Institute e.V. (ASI), Berufsverband Deutscher Markt- und Sozialforscher e.V. (BVM)] which were implemented to improve the German federal law to protect the ethical rights of individuals. These guidelines specify amongst others how the person has to be approached and treated in the interview and how the personal as well as the collected data has to be handled and stored. The application of the guidelines secures that the studies follow the valid ethical standards. Therefore, additional ethical approval is not necessary. The study presented here was approved according to the guidelines of the German professional institution of social researchers.

### Instruments

The EMBU by Perris and colleagues [8] was chosen to establish an economical German instrument for recalled parental rearing practices since it comprises good internal reliability (> .70), is a well implemented questionnaire and appears to be generalizable across cultures [17]. The questionnaire Fragebogen zum erinnerten elterlichen Erziehungsverhalten (FEE [11]) [Recalled Parental Rearing Behavior] represents the only German short version of the EMBU for measuring recalled parental rearing behavior which is not identical to any existing English EMBU short versions. Since the factor loadings of the English and the German long versions of the EMBU differ considerably, the item selection for this German short version (FEE) was based on the eight highest factor loadings on the scales of the German long version [12]. For the scale "emotional warmth" the items 2, 7, 9, 12, 14, 15, 17, 24, for the scale "rejection/punishment" the items 1, 3, 6, 8, 16, 18, 20, 22 and for the scale "control/overprotection" the items 4, 5, 10, 11, 13, 21, 23 were selected. Even though the two English versions and the German short version of the EMBU [3,10] care based on different item selections, the same theoretical constructs (emotional warmth, rejection/punishment, control/overprotection) could be identified for the English and the German versions.

The FEE includes 24 items to be answered separately for both, mother and father, on a Likert-type scale with the categories 1 (no, never), 2 (yes, sometimes), 3 (yes, often) and 4 (yes, always) (range = 8–32, for an example of the items see [8]). This separate answering style had been evaluated successfully for the original version of the EMBU by a principal component factor analysis [8]. The scales of the FEE were factor analytically derived as in the English versions [9]: emotional warmth, rejection/punishment, control/overprotection. A high score on the scale "rejection" means highly rejecting and punishing recalled parental rearing behavior. Similarly, for emotional warmth and overprotection high scores indicate great emotional warmth and overprotective recalled parental rearing behavior.

General and specific life satisfaction was assessed by the Fragebogen zur Lebenszufriedenheit (FLZ) [Life Satisfaction Questionnaire] by Fahrenberg, Myrtek, Schumacher and Brähler [18]. This instrument measures the following areas of life satisfaction: health, job and profession, finances, leisure, spouse/partner, relationship to own children, self, sexuality, friends and relatives, and home. All of the ten subscales of the FLZ (with seven items each) were rated from 1 (very dissatisfied) to 7 (very satisfied). High scores indicate high satisfaction with the areas of life. The reliability ranged from .82 to .95.

The Inventory of Interpersonal Problems (IIP) [19] assesses self-reported difficulties in social interaction. The German version of the instrument (IIP-D) [20] includes 64 items subdivided into eight subscales (overly domineering; overly vindictive; overly cold; overly socially avoidant; overly non-assertive; overly exploitable; overly nurturant; overly intrusive). The items were rated on a five-point Likert scale from 0 (not at all) to 4 (very much). High scores on the scales of the IIP stand for great difficulties in social interactions. The reliability of the scales ranges from .36 to .64.

### Statistical analysis

The statistical data analysis was performed by means of SPSS for Windows version 10.0.

Concerning the psychometric properties of the FEE scales the internal consistency of the scales was specified by Cronbach's alpha. In addition, a split-half reliability was calculated for each scale by a Spearman-Brown correlation. Since the extraction of the items is based on the factor loadings of the German long version, the internal reliability of the original and the new German short version may be similar.

In order to test the three-dimensional structure and the factorial validity of the FEE a principal component analysis (PCA) was calculated separately for the mother and the father. Hereby, an orthogonal varimax rotation was applied ahead of time. The factorial structure for the English and the German long version had already been replicated in several studies [9]. Therefore, the original factorial structure can be expected to be replicable also for the new German short version.

Differences between the mother- and father-scale ratings were calculated by t-tests for paired samples. Since parental rearing behavior proved to be specific to the parent's gender [8], significant differences between the mother- and father-scales were to be expected. However, the similar constructs between the mother and the father might be associated with higher than opposing constructs between the mother and the father rating. Furthermore, the scales were correlated with each other by using a Pearson correlation coefficient (two-tailed).

To specify the influence of age (three categories: 18–30, 31–60 and 61–92 yrs.) and gender on parental rearing behavior, two-factorial univariate analyses of variance were calculated. T-tests were calculated to evaluate the effect of the child's gender (child gender) on the rated parent. Furthermore, the interaction effects between the gender of the evaluated parent (father/mother) and the gender of the child were calculated by a two-factorial multivariate analysis of variance. It can be postulated that older subjects may recall more rejection and stricter parental rearing behavior than younger ones [4], whereby the male subjects may perceive more rejecting parental rearing behavior than the female ones.

To specify the construct validity of the FEE, the well established associations between perceived parental rearing behavior and life satisfaction as well as interpersonal problems were tried to be replicated using the FEE. Pearson correlation coefficients (two-tailed) were calculated to examine associations between the FEE and other constructs. Hereby, non-supportive, rejecting and overprotective parental rearing behavior may be connected to more interpersonal problems and less life satisfaction than in the case of supportive, non-rejecting and non-overprotective parental rearing behavior.

## Results

### Reliability of the FEE

The means and standard deviations of the FEE scales and some additional characteristics are depicted in Tables 1 and 2. Each of the three FEE scales yielded a good to satisfactory internal consistency and split-half reliability for both paternal and maternal rearing behavior. For all three scales, significant mean differences between the experience of recalled maternal vs. paternal rearing behavior emerged in terms of less rejecting and less punitive, emotionally warmer and more overprotective mothers. The highest score on emotional warmth had 0.1 percent of the sample whereas rejection had 0.2 percent and overprotection reported 0.02 percent of the participants. The lowest score on emotional warmth had 0.6 percent, rejection described 7.4 percent and overprotection experienced 1.4 percent of the participants.

**Table 1:** Psychometric properties of the FEE scales

Scales		M	SD	α	r_tt_	Skewness	Excess
Rejection and punishment	paternal N = 2771	12.30	4.25	.89	.88	1.45	2.30
	maternal N = 2871	11.88	3.94	.87	.86	1.57	2.69

Emotional Warmth	paternal N = 2771	18.73	4.55	.86	.86	0.04	0.02
	maternal N = 2871	21.03	4.41	.86	.86	-0.20	-0.05

Control and overprotection	paternal N = 2771	14.59	3.87	.74	.73	0.62	0.32
	maternal N = 2871	15.04	3.82	.72	.70	0.50	0.11

M ... mean

SD ... standard deviation

α ... internal consistency (Cronbach's alpha)

r_tt _... split-half-reliability (Spearman-Brown)

**Table 2:** Comparison of the paternal and maternal FEE scales

Scales		t	p(t)	d
Rejection and punishment	paternal N = 2771	7.02	.001	0.107
	maternal N = 2871			

Emotional Warmth	paternal N = 2771	-35.58	.001	0.513
	maternal N = 2871			

Control and overprotection	paternal N = 2771	-9.63	.001	0.117
	maternal N = 2871			

Notes: significance of mean differences between perceived paternal

p(t)... and maternal rearing (t-test for paired samples)

d ... effect sizes

### Factorial validity of the FEE

Furthermore, the factorial structure as well as the independence of the three dimensions were tested. In Table 3 the factor loadings for the recalled parental rearing items are depicted. The original factor structure could be satisfactorily replicated (with the exception of item 13 – "Did your parents use the expression: If you don't do this, then I'll be sad"). The largest proportion of variance was explicable by the factor "rejection and punishment" (19.5% for the paternal items and 18.0% for the maternal items) was followed by "emotional warmth" (17.6% and 17.10%, respectively) and "control and overprotection" (11.5% for both factors). The cumulative explained variance was 48.6% for recalled paternal rearing and 46.6% for recalled maternal rearing behavior.

**Table 3:** Factor structure of perceived parental rearing according to FEE (principal component analysis with varimax rotation and extraction of predefined three factors)

	Paternal rearing (N = 2.771)				Maternal rearing (N = 2.871)			
		Factor loadings				Factor loadings		

Item-No.	h^2^	I	II	III	h^2^	I	II	III

Scale I: Rejection and punishment								
								
1	.65	.76	-.14	.23	.61	.72	-.19	.23
3	.38	.58	-.08	.18	.36	.56	-.08	.18
6	.50	.68	-.11	.15	.50	.66	-.15	.20
8	.63	.74	-.18	.20	.61	.72	-.19	.21
16	.57	.72	-.13	.18	.57	.71	-.15	.19
18	.65	.79	-.11	.12	.62	.77	-.13	.12
20	.49	.68	-.16	.11	.48	.67	-.12	.09
22	.56	.69	-.16	.24	.51	.64	-.18	.26
								
Scale II: Emotional warmth								
								
2	.54	-.20	.71	-.07	.55	-.15	.71	-.12
7	.47	-.08	.68	.06	.46	-.15	.66	.11
9	.42	-.22	.61	.07	.42	-.17	.62	.02
12	.57	-.10	.75	-.01	.57	-.05	.75	-.01
14	.51	-.03	.71	.05	.49	-.08	.70	.07
15	.56	-.03	.75	.03	.54	-.19	.70	.12
17	.54	-.20	.71	-.07	.55	-.14	.72	-.08
24	.48	.01	.69	.01	.47	-.08	.67	.12
								
Scale III: Control and overprotection								
								
4	.45	.05	.04	.67	.37	.13	.03	.59
5	.41	.19	.08	.61	.41	.09	.05	.63
10	.41	.35	-.14	.52	.40	.36	-.16	.50
11	.47	.19	.06	.65	.41	.28	.05	.57
13	.31	.46	.22	.23	.26	.23	.15	.42
19	.43	.27	-.17	.57	.35	.23	-.12	.54
21	.34	.15	-.05	.56	.25	.25	.01	.43
23	.32	.15	.19	.51	.45	-.03	.16	.65

% variance		19.5	17.6	11.5		18.0	17.1	11.5

Notes:h^2 ^= communality;

factor loadings > .40 are underlined

Eigen values of the unrotated solutions:

paternal rearing: Faktor I 6.44/Faktor II 3.77/Faktor III 1.46

maternal rearing: Faktor I 6.29/Faktor II 3.61/Faktor III 1.29

Factor I = Rejection and punishment

Factor II = Emotional warmth

Factor III = Control and overprotection

### Intercorrelations of the FEE subscales

Table 4 shows that some of the scales are highly correlated. The highest intercorrelations could be found between the identical (regarding content) scales for fathers and mothers (r = .70 to .77). Positive but moderate correlations were also found when investigating one parent or both parents concerning the scales "rejection and punishment" as well as "control and overprotection". Furthermore, the scale "emotional warmth" correlated negatively with the scale "rejection and punishment" whereas "emotional warmth" and "control and overprotection" were unrelated.

**Table 4:** Intercorrelations of the FEE scales

	Scales	1	2	3	4	5	6
1	Paternal rejection and punishment		-.31*	.54*	.70*	-.27*	.43*
2	Paternal emotional warmth			-.01	-.19*	.71*	.01
3	Paternal control and overprotection				.45*	.02	.77*
4	Maternal rejection and punishment					-.36*	.53*
5	Maternal emotional warmth						.02
6	Maternal control and overprotection						

Notes: Pearson correlations (two-tailed), *p < .001

2.768 ≤ N ≤ 2.871

### Influence of socio-demographic factors

In addition, the influence of age and gender on recalled parental rearing behavior was calculated. Age (three categories: 18–30, 31–60 and 61–92 yrs.) exerted a significant influence on the perception of recalled paternal rejection and punishment as well as on emotional warmth. Furthermore, the age also influenced the father's recalled rejection and punishment as well as the mother's emotional warmth (see Tables 5 and 6). According to their memories, the older the subjects, the stronger they experienced their parents' rejection and the less their parents' emotional warmth. In addition, they reported much stricter and less emotionally warm parental rearing behavior in reference to their father as well as a lack of emotional warmth from their mother.

**Table 5:** The influence age on the recalled parental rearing

	Age groups					
	18–30	31–60	61–92	F	df	p(F)

	M (SD)	M (SD)	M (SD)			

Parental rejection and punishment	11.72 (3.71)	12.17 (3.75)	12.23 (3.83)	3.71	2/2765	.02
Parental emotional warmth	20.52 (4.25)	19.69 (4.14)	19.73 (3.97)	9.13	2/2765	.00
Father's rejection and punishment	11.83 (4.17)	12.41 (4.25)	12.48 (4.28)	4.75	2/2768	.01
Mother's rejection and punishment	11.66 (3.96)	11.91 (3.93)	11.97 (3.96)	1.16	2/2868	.32
Father's emotional warmth	19.33 (4.80)	18.58 (4.52)	18.55 (4.37)	6.57	2/2768	.00
Mother's emotional warmth	21.69 (4.41)	20.83 (4.48)	20.90 (4.23)	8.57	2/2868	.00

M ... mean

SD ... standard deviation

p(F)... significance of mean differences between the age groups (ANOVA)

**Table 6:** The influence child's gender on the recalled parental rearing

	Child's gender					
	female	male	d	t	df	p(t)

	M (SD)	M (SD)				

Parental rejection and punishment	11.85 (3.73)	12.39 (3.79)	-.14	3.79	1/2766	.00
Parental emotional warmth	20.07 (4.28)	19.63 (3.93)	-.11	-2.81	2703.97	.00
Father's rejection and punishment	11.91 (4.14)	12.81 (4.33)	-.21	5.59	1/2769	.00
Mother's rejection and punishment	11.79 (3.97)	11.98 (3.92)	-.05	1.27	2869	.21
Father's emotional warmth	19.05 (4.70)	18.32 (4.34)	-.16	4.22	2702.71	.00
Mother's emotional warmth	21.08 (4.53)	20.96 (4.26)	-.04	-.74	2869	.46

M ... mean

SD ... standard deviation

d ... effect sizes

p(t)... significance of mean differences between perceived paternal and maternal rearing (t-test for paired samples)

The data also revealed significant main gender effects on recalled parental rearing behavior, i.e. the male subjects recalled more rejecting and stricter parental rearing behavior than the female subjects. In addition, the parents' emotional warmth was less remembered by the male subjects than by the female subjects. To be specific, the female subjects reported their fathers as emotionally warmer and less punishing than did the male subjects (see Tables 5 and 6).

Furthermore, there was a significant interaction effect between the gender of the evaluated parent (father/mother) and the gender of the child concerning "rejection and punishment" (M_males _= 12.81 (SD = 4.33) vs. M_females _= 11.91 (SD = 4.12); F = 30.32; df = 1/2769; p(F) < .01) and "emotional warmth" (M_males _= 18.32 (SD = 4.34) vs. M_females _= 19.05 (SD = 4.71); F = 16.65; df = 1/2766; p(F) < .01). Significant interactive effects between age and gender could not be observed.

### Correlations between the FEE and other scales

As a first step for evaluating the external validity of the FEE its relationships to other scales were tested (see Tables 7 and 8 for p < .001). Concerning life satisfaction (FLZ), low to satisfactory relationships were established to most of the examined areas from .07 to .39: Subjects who had recalled more rejecting, punitive and controlling parental rearing behavior and a lack of emotional warmth reported a lower level of life satisfaction than subjects with a more positive rearing experience.

**Table 7:** Correlations between perceived paternal rearing and both life satisfaction (FLZ) and interpersonal problems (IIP)

	FEE scales		
	Paternal rearing		
	Rejection and punishment	Emotional warmth	Control and over-protection
FLZ health	-.21*	.16*	-.11*
FLZ job and profession	-.11*	.17*	.01
FLZ finance	-.13*	.13*	-.03
FLZ leisure	-.09*	.08*	-.03
FLZ spouse/partner	-.23*	.16*	-.12*
FLZ children	-.34*	.22*	-.20*
FLZ self	-.27*	.21*	-.15*
FLZ sexuality	-.17*	.14*	-.08*
FLZ friends/relatives	-.26*	.24*	-.12*
FLZ home	-.19*	.07*	-.11*
FLZ general life satisfaction	-.26*	.21*	-.13*
			
IIP-BC (overly vindictive)	.37*	-.18*	.26*
IIP-DE (overly cold)	.33*	-.20*	.22*
IIP-FG (overly soc. avoidant)	.31*	-.17*	.22*
IIP-HI (overly non-assertive)	.19*	-.13*	.12*
IIP-JK (overly exploitable)	.19*	-.09*	.16*
IIP-LM (overly nurturant)	.19*	-.03	.19*
IIP-NO (overly intrusive)	.32*	-.06	.28*
IIP-PA (overly domineering)	.39*	-.12*	.32*

Notes: Pearson correlations (two-tailed), *p < .001; 2.651 ≤ N ≤ 2.820

**Table 8:** Correlations between perceived maternal rearing and both life satisfaction (FLZ) and interpersonal problems (IIP)

	FEE scales		
	Maternal rearing		
	Rejection and punishment	Emotional warmth	Control and over-protection
FLZ health	-.23*	.19*	-.12*
FLZ job and profession	-.08*	.16*	.01
FLZ finance	-.13*	.14*	-.05
FLZ leisure	-.11*	.11*	-.05
FLZ spouse/partner	-.28*	.20*	-.12*
FLZ children	-.30*	.19*	-.20*
FLZ self	-.29*	.23*	-.17*
FLZ sexuality	-.17*	.13*	-.09*
FLZ friends/relatives	-.24*	.21*	-.15*
FLZ home	-.20*	.07*	-.14*
FLZ general life satisfaction	-.28*	.22*	-.15*
			
IIP-BC (overly vindictive)	.36*	-.20*	.29*
IIP-DE (overly cold)	.33*	-.24*	.24*
IIP-FG (overly soc. avoidant)	.32*	-.20*	.22*
IIP-HI (overly non-assertive)	.19*	-.16*	.14*
IIP-JK (overly exploitable)	.22*	-.12*	.19*
IIP-LM (overly nurturant)	.21*	-.03	.21*
IIP-NO (overly intrusive)	.35*	-.09*	.29*
IIP-PA (overly domineering)	.41*	-.16*	.34*

Notes: Pearson correlations (two-tailed), *p < .001; 2.651 ≤ N ≤ 2.820

Low to satisfactory relationships to recalled parental rearing, behavior, in particular on the scales "rejection and punishment" and "control and overprotection", also appeared for interpersonal problems (IIP factors) with a range of .09 to .39: Subjects who recalled rejective and strict rearing behavior spoke of difficulties in trusting other people, supporting others and caring for others' needs. Furthermore, they had problems cooperating, and they attempted to dominate and control others. They also described themselves as resentful and quarrelsome.

## Discussion

Based on the data presented in this paper the FEE represented a reliable and valid instrument for the assessment of adults' memories of their parents' rearing behavior for the German-speaking areas. Its relatively low number of items allowed for an economical application in research and clinical work. Both, the items and the three scales showed satisfactory to good psychometric properties. The reliability figures highly corresponded to the values obtained for the original long version of the EMBU from 14 countries (N = 3.500) [21].

In the present data, the results from the principal component analysis successfully replicated the previously obtained factorial structure of the EMBU [21]. The cumulative variance explained by the three factors is higher than the corresponding figures for the EMBU [21] and confirmed the factorial validity of the FEE. Also, the intercorrelational pattern of the FEE factors was consistent with previous results using the original long version of the EMBU [21].

When comparing recalled paternal and maternal rearing behavior, a number of significant differences were found, whose relevance deserves a closer look due to the small numerical mean differences. However, the findings mothers compared to fathers tended to be emotionally warmer and less rejecting on the one hand and more controlling and overprotective on the other, were in agreement with the results of the study by Gerlsma and Emmelkamp [22].

Whereas based on the EMBU only a few studies reported age and gender effects, the presented data indicated some influence of these factors on recalled parental rearing behavior. Based on their memories, older subjects recalled their parents as more rejecting and less emotionally warm than did their younger counterparts. This age effect might be explained by the historical changes in parenting attitudes and behavior in child rearing practices. In addition, the historical German background may also have to be considered. Furthermore, based on their memories, the female subjects reported having received more emotional warmth from their fathers than the male subjects did, whereby the latter recalled their fathers as stricter and more rejecting.

In the literature a few studies have focused on the connection between life satisfaction and parental rearing behavior [2,3,23,24]. In line with the literature, the presented data have shown that recalled parental rearing behavior was connected to life satisfaction and self-reported interpersonal problems.

The retrospective assessment of recalled parental rearing behavior represents a specific problem in assessing the actual parental rearing experienced during childhood or its subjective representation [25,26]. The subjective representation may reflect the present mood, errors in autobiographical memory (un-/conscious distortions), false memories or idiosyncratic reconstructions of the subjects' personal history. However, the existing literature did not provide consistent and conclusive data on the mood-congruent recall of relevant personal stimuli [25,27-30] as well as on the validity of retrospective data on parental rearing behavior [31]. Therefore, longitudinal studies with independent raters should be considered for the validity of parental rearing practices (see [32]). Unfortunately, in clinical practice, the child rearing experienced by patients can only be assessed retrospectively after the onset of the disorder. Nevertheless, the then obtained information can be of help in the therapeutic process.

Based on the data from a representative sample, general conclusions cannot be drawn, since the large sample size could easily lead to significant results, e.g. a correlation coefficient of r = .07 (p < .001). In the context of validating the instrument, this study did not focus on any connection to psychological symptoms. Therefore, additional studies that include assessments of psychological symptoms as well as a formal comparison of the German long and short version of the EMBU as well as the original version would still be essential. Furthermore, retest-reliability testing and connections to other parental child rearing questionnaires or external ratings would be helpful to further specify the validity and reliability of this short version.

## Conclusion

In summary, the afore-discussed data show that the FEE is a reliable and valid instrument for the retrospective assessment of subjective representations of parental rearing behavior. Moreover, this information will be relevant not only to the research concerning psychological disorders but also to the field of non-clinical applications.

## Competing interests

The authors declare that they have no competing interests.

## Authors' contributions

KP did the final draft of the manuscript and critically revised it for the intellectual content. HB and SS have given final the approval of the version to be published. JS substantially contributed to the analysis and the interpretation of the data. He wrote the first draft of the manuscript. AH substantially contributed to the analysis and the interpretation of data. EB was responsible for the collection of the data and the general supervision of the research group. He substantially contributed to the conception and the design of the study as well as the acquisition of the data. All authors read and approved the final manuscript.

## Pre-publication history

The pre-publication history for this paper can be accessed here:

http://www.biomedcentral.com/1471-2288/9/17/prepub
